# Screening and functional identification of lncRNAs in antler mesenchymal and cartilage tissues using high-throughput sequencing

**DOI:** 10.1038/s41598-020-66383-1

**Published:** 2020-06-11

**Authors:** Dan-yang Chen, Ren-feng Jiang, Yan-jun Li, Ming-xiao Liu, Lei Wu, Wei Hu

**Affiliations:** College of Life Science, Jilin Agriculture University, Changchun, Jilin Province 130118 China

**Keywords:** Biochemistry, Biotechnology, Computational biology and bioinformatics, Molecular biology

## Abstract

Long non-coding RNA (lncRNA) is a transcription product of the mammalian genome that regulates the development and growth in the body. The present study aimed to analyze the expression dynamics of lncRNA in sika antler mesenchymal and cartilage tissues by high-throughput sequencing. Bioinformatics was applied to predict differentially expressed lncRNAs and target genes and screen lncRNAs and mRNAs related to osteogenic differentiation, cell proliferation, and migration. Finally, the expression of the lncRNAs and target genes were analyzed by qRT-PCR. The results showed that compared to the cartilage tissue, the transcription levels of lncRNA and mRNA, 1212 lncRNAs and 518 mRNAs, in mesenchymal tissue were altered significantly. Thus, a complex interaction network was constructed, and the lncRNA-mRNA interaction network correlation related to osteogenic differentiation, cell proliferation, and migration was analyzed. Among these, the 26 lncRNAs and potential target genes were verified by qRT-PCR, and the results of qRT-PCR were consistent with high-throughput sequencing results. These data indicated that lncRNA promotes the differentiation of deer antler mesenchymal tissue into cartilage tissue by regulating the related osteogenic factors, cell proliferation, and migration-related genes and accelerating the process of deer antler regeneration and development.

## Introduction

Sika deer antler is the only bone organ that can be completely regenerated in mammals. It has an incomparable growth rate and super stability which could not be exceeded by other animals. Thus, it is an ideal model for studying organ regeneration and wound repair^1^. Deer antler completes periodic regeneration through cartilage osteogenesis and a combination of several factors. The initial stage of antler cartilage formation is due to the rapid growth of mesenchyme. This mesenchyma differentiates into cartilage, which ossifies into the bone and promotes antler regeneration^2^.

Due to the complexity of antler development and regeneration, many factors affect the development and regeneration of deer antler. Among these, some coding and non-coding RNAs play a major role in the regeneration of deer antler^3^. In a previous study, the high-throughput sequencing of miRNAs was performed in the mesenchymal and cartilage tissues of the antler tip. Also, the miR-18a, miR-18b, miR-1, miR-let-7a, miR-let-7f, miR-20b-5p, miR-93-5p, miR-15a, miR-15b, miR-19a, miR-19b, miR-106b, and miR-148a related to the differentiation and proliferation of antler cells were verified^4–6^. Therefore, in order to further study the mechanism of regeneration of deer antler, we explored the role of non-coding RNA in the rapid growth of deer antler.

Long non-coding RNA (lncRNA) is a newly discovered type of non-coding RNA with a length >200 nucleotides, accounting for 80% of the non-coding RNA^7^. Reportedly, some lncRNAs play key roles in regulating gene expression through multiple mechanisms in various biological processes, such as cell proliferation and differentiation, apoptosis, inflammatory response, and oxidative stress^8^. In recent years, several abnormalities were detected in the lncRNA expression in various tumors, osteoporosis, and heart-related diseases^9–11^. A large number of studies have shown that lncRNAs regulate the differentiation and function of cartilage. It has been confirmed that lncRNAs affect the differentiation of mesenchymal cells, as well as regulate the osteogenesis of mesenchymal cells^12^. Gao *et al*. found that the expression level of lncRNA MALAT1 decreased in mesenchymal stem cells of patients with osteoporosis. When MSCs were treated with osteogenic induction, the expression of MALAT1 increased. After siRNA mediated gene knockdown, the osteogenic differentiation of mesenchymal stem cells was significantly reduced, indicating that lncMALAT1 could be a positive regulator of osteogenic differentiation^13^. Zhang *et al*. proved that the lncRNA DANCR in human bone marrow-derived mesenchymal stem cells (HBMSC) was significantly reduced during osteogenic differentiation. The cells were transfected with DANCR-targeting siRNA (siDANCR); consequently, the gene knockdown enhanced the proliferation of HBMSCs and osteogenic differentiation, indicating that lncRNA DANCR plays a major role in osteogenesis^14^.

Therefore, we speculated that lncRNAs are crucial in the mesenchymal differentiation of antler tip tissue into cartilage tissue. However, only a few studies have reported the role of lncRNAs in antler mesenchymal and cartilage tissue development. In this study, high-throughput sequencing technology was used to investigate the expression profiles of lncRNA in mesenchymal and cartilage tissues of deer antler and predict the functions of these lncRNAs through bioinformatics analysis. In addition, the identification and analysis of lncRNAs and mRNAs related to osteogenic differentiation, proliferation, and migration of antler tissue were performed to provide the theoretical basis for further research.

## Results

### High-throughput sequencing

The total RNA from six samples of mesenchyma and cartilage was sequenced with Illumina Novaseq. 6000; each produced >90 million reads. The original fragments were filtered to obtain clean reads. Then, HISAT2 2.1.0 software was used to map the total clean reads to the genome; the proportion of pure reads mapped to the genome was >97%. In order to evaluate the quality of the RNA-seq data, FastQ 0.11.2 software was used to obtain the quality score (Q) for each base assigned. The sequencing Q30 of each sample was>95%, indicating good sequencing quality (Table 1). Finally, the genes and transcripts were assembled according to the result of reads comparison. Also, the expressed genes and transcripts were annotated by synthesizing multiple databases (ENSEMBL, NCBI, Uniprot, GO, KEGG) based on the selected reference genome.

**Table 1:** Data filter statistics and mapping rate.

Sample name	Raw reads number	Raw bases	Clean reads number	Clean bases	Clean rate (%)	Q30 (%)	Total reads	Mapped reads	Uniquely mapped reads	Multiple mapped reads
C1	107976268	16196440200	105843120	15847150908	97.84	95.74	105843120 (100.00%)	829724877 (78.39%)	78371374 (94.45%)	4601113 (5.55%)
C2	102655950	15398392500	100535076	15051874722	97.75	95.68	100535076 (100.00%)	76775043 (76.37%)	72768941 (94.78%)	4006102 (5.22%)
C3	94191664	14128749600	92065762	13785172696	97.57	95.5	92065762 (100.00%)	66297518 (72.01%)	62685998 (94.55%	3611520 (5.45%)
M1	107352654	16102898100	105336702	15773218776	97.95	95.9	105336702 (100.00%)	81907897 (77.76%)	78242379 (95.52%)	3665518 (4.48%)
M2	107094318	16064147700	104743846	15677865434	97.6	95.66	104743846 (100.00%)	80056882 (76.43%)	75651498 (94.50%)	4405384 (5.50%)
M3	106770634	16015595100	104407040	15633725952	97.62	95.63	104407040 (100.00%)	74612317 (71.46%)	71599459 (95.96%)	3012858 (4.04%)

### Identification and characterization of lncRNAs

**Figure 1:**
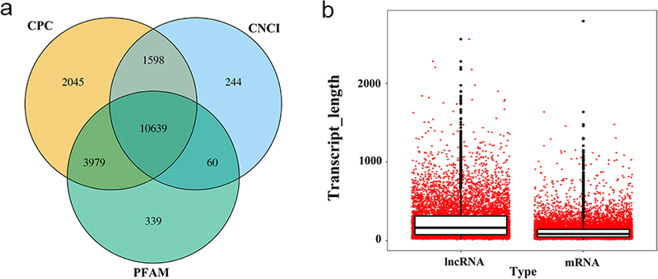
Identification of lncRNA. (**a)** CPC, CNCI, and HMMER were used to predict the encoding ability of unknown transcripts, and 10639 novel lncRNAs were identified. (**b**) The length distribution of lncRNA and the mRNA transcripts. Most of the lncRNAs are longer than mRNA.

Based on the reference genome, 156 known lncRNAs were found. For the unknown transcripts assembled, the coding capabilities were predicted by CPC 0.9r2, CNCI and HMMER 3.1b1. The results showed that a total of 10639 novel lncRNAs were found after screening by the three software (Fig. 1a). In terms of length, most of the lncRNAs were relatively longer as compared to the mRNA. The lncRNA was mainly around 1500 bp, and the mRNA was primarily around 750 bp (Fig. 1b).

### Analysis of the differentially expressed lncRNA and mRNA in mesenchymal and cartilage tissues

**Figure 2:**
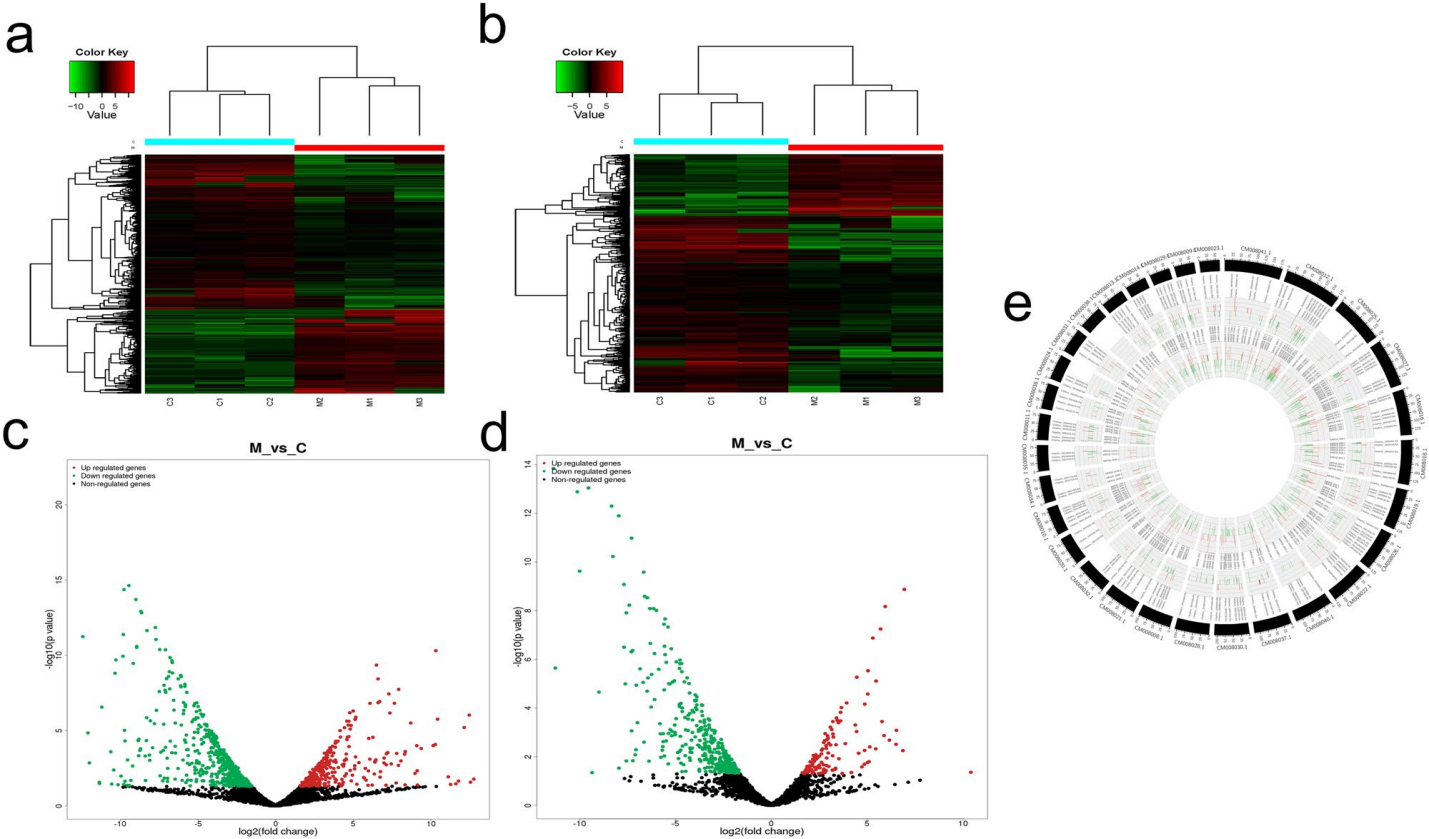
Differentially expressed lncRNA and mRNA revealed by high-throughput sequencing between mesenchymal and cartilage tissues. (**a**,**b**) The clustering heatmaps of the differential lncRNA and mRNA, C1, C2, C3: Cartilage tissue of the three sika deer antler, M1, M2, M3: Mesenchymal tissue. (**c**,**d**) Volcano diagrams of differential lncRNA and mRNA. The abscissa and ordinate represent X = log2 (multiple changes) and Y = −log10 (P-value), the red dots represent upregulated differential genes, the green dots represent downregulated differential genes, and the black dots represent genes with no significant difference. **e:** Distribution of differential lncRNAs and mRNAs in 26 sika deer chromosomes. Circos map was drawn by Circos 0.67-7 software. The periphery is the reference genome, followed by mRNA, the innermost circle is lncRNA, red indicates upregulation, and green indicates downregulation.

The DEseq. 1.28.0 software was used for differential expression analysis of lncRNA and mRNA between two tissues. The screening criteria were |log_2_FC | ≥1 and P-value ≤0.05. Compared to the cartilage tissue, there are a total of 1212 lncRNA and 518 mRNA changes in the mesenchyma. A clustering heatmap was used to show the difference in the expression levels of the lncRNA and the mRNA among samples (Fig. 2a,b). According to the fold-change value, the differential lncRNAs and mRNAs were divided into upregulated lncRNAs (427) and mRNAs (134) (fold-change ≥2) and downregulated lncRNAs (785) and mRNAs (384) (fold-change ≤0.05) (Fig. 2c,d). These results showed that the differences in the expression of lncRNA and mRNA in mesenchymal and cartilage may play a major role in the rapid growth of the antler.

The Circos map of differentially expressed lncRNAs and mRNAs were drawn by the Circos 0.67-7 software (Fig. 2e). The outer ring is the reference genome, followed by mRNA, and the innermost circle is lncRNA. Red indicates upregulation, and green indicates downregulation. We found that the lncRNAs and mRNAs were evenly distributed on 26 chromosomes without obvious position preference. Based on the number analysis of the differentially expressed genes, the number of changes in lncRNA was greater than the number of changes in the mRNA as compared to the mRNA Circos map. The same mRNA can be regulated by multiple lncRNAs, and the same lncRNA can regulate different mRNAs. The differential expression of lncRNA is closely related to the differential changes in mRNA, indicating that the interaction between lncRNA and mRNA affects the rapid growth of the antler.

### GO and KEGG analysis

**Figure 3:**
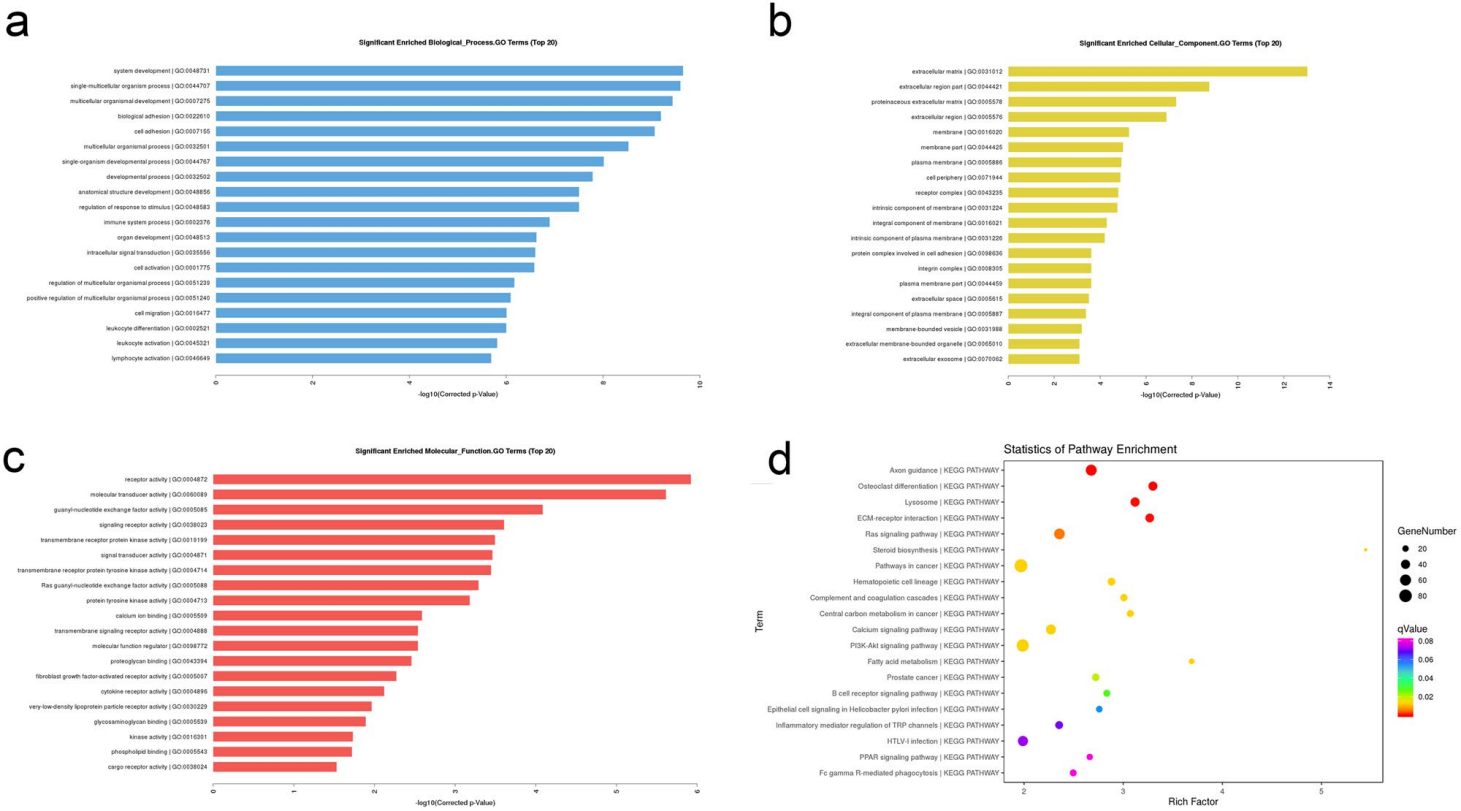
GO and KEGG analysis of target genes. (**a–c**) The first 20 GO terms of target genes in biological process, cellular component, and molecular function. (**d**) KEGG pathway analysis of the target gene. The results show about 20 pathways with significant functional changes.

In order to analyze the rapid growth process of antler, the differential expression genes were analyzed by GO enrichment and KEGG pathways. The enrichment of target genes in the GO project includes 1021 biological processes, 121 molecular functions, and 82 cellular components (P < 0.05), mainly involving system development, single-multi cellular organism process, multicellular organismal development and organ development, intracellular signal transduction, cell activation, cell migration, and cell differentiation. (Fig. 3a–c) Information on the above Go terms is listed in Supplementary Table S1a. The KEGG analysis of differential expression genes showed statistically significant functional changes in 57 pathways (P < 0.05). According to the KEGG analysis, these differential genes were mainly concentrated in some signaling pathways related to bone formation, cell proliferation and migration, including axon guidance, osteoclast differentiation, hematopoietic cell lineage, Ras signaling pathway, calcium signaling pathway, and PI3K-Akt signaling pathway (Fig. 3d) Information on the above signaling pathways is listed in Supplementary Table S1b. The GO and KEGG analysis showed that bone formation, cell proliferation, and migration might be some of the reasons for the rapid growth of the antler. Therefore, we speculated that the lncRNAs promote osteogenic differentiation, cell proliferation, and migration by regulating the related genes, and thus, indirectly regulating the antler growth and development.

### Co-differential expression of lncRNA and potential target genes

**Figure 4:**
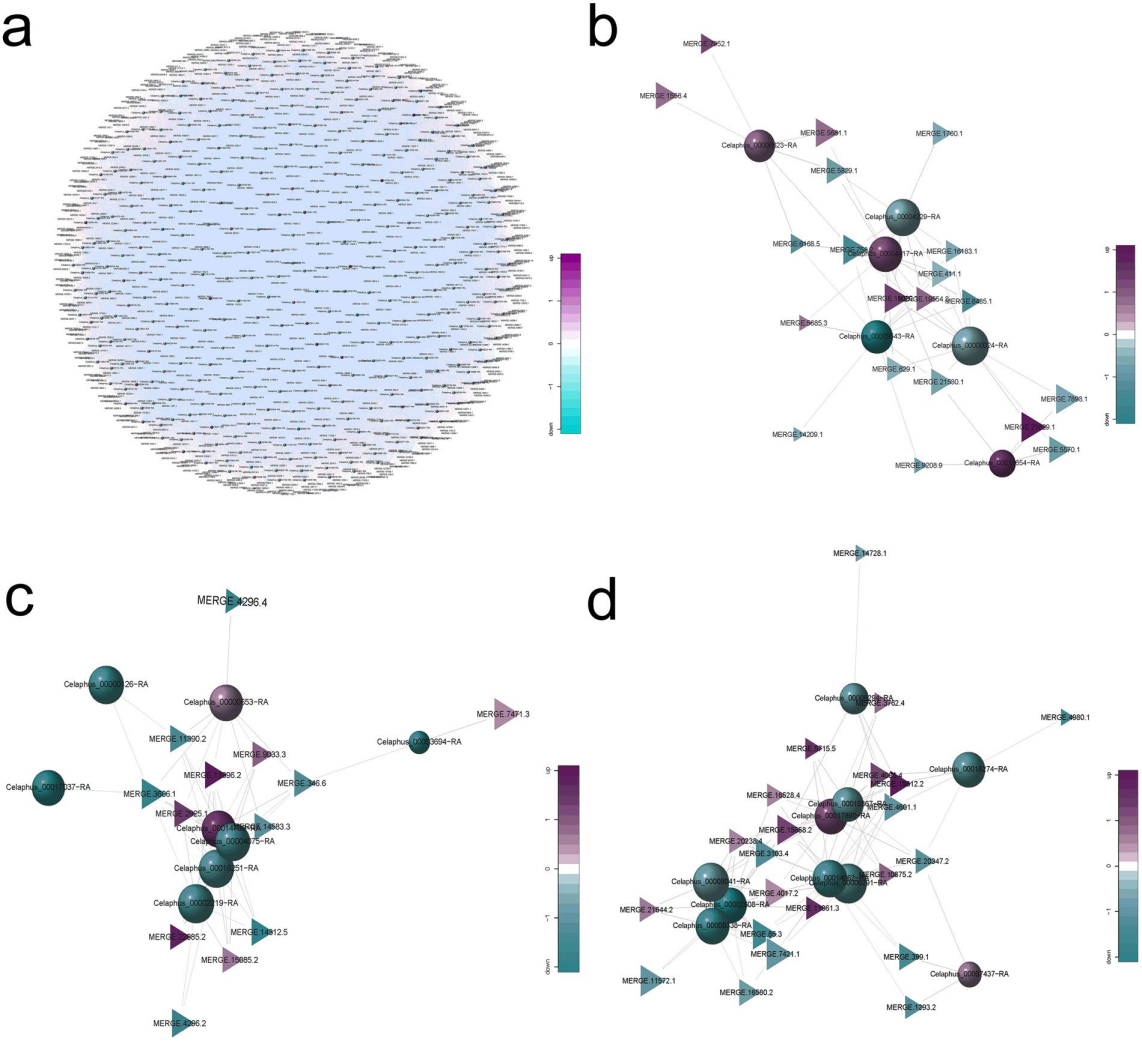
Regulatory networks of lncRNAs and target genes. (**a**) All lncRNA and target gene regulatory networks. (**b–d**) The regulatory network of osteogenic differentiation, cell proliferation, and migration-related target genes and lncRNA. The balls indicate target genes, and triangles indicate lncRNA. The blue ball indicates downregulation, and purple ball indicates upregulation. The lines represent the interactions between diverse RNAs.

The regulatory network map was constructed, which revealed 28989 pairs of lncRNA-mRNA, of which 17875 pairs were positively regulated and 11114 pairs were negatively regulated. The co-expression network is shown in Fig. 4a. The MCL v2.0 software was used to mine the sub-modules of the total network, and the mRNA-lncRNA interaction pairs related to antler development were screened to construct the common regulatory network. The interaction diagram of 20 lncRNAs and 6 mRNAs with osteogenesis-related genes (Fig. 4b), the 13 lncRNAs and 8 mRNAs related to cell proliferation (Fig. 4c), and 22 lncRNAs and 10 mRNAs related to cell migration was constructed (Fig. 4d). The information on the above genes is listed in Supplementary Table S2a–c.

### Screening and qRT-PCR verification of genes and lncRNAs related to osteogenic differentiation, cell proliferation, and migration

Five lncRNAs were shown in the regulatory network of the osteogenic genes: MERGE.13896.2, MERGE.15085.2, MERGE.3606.1, MERGE.14583.3, and MERGE.14312.5. Moreover, this regulatory network displayed that four genes, RCAN3 (Celaphus_00014788), DEF8 (Celaphus_00004075), TCEA1 (Celaphus_00016251), CSF1 (Celaphus_00002219), related to osteogenic differentiation may be regulated collectively by the five lncRNAs.

**Figure 5:**
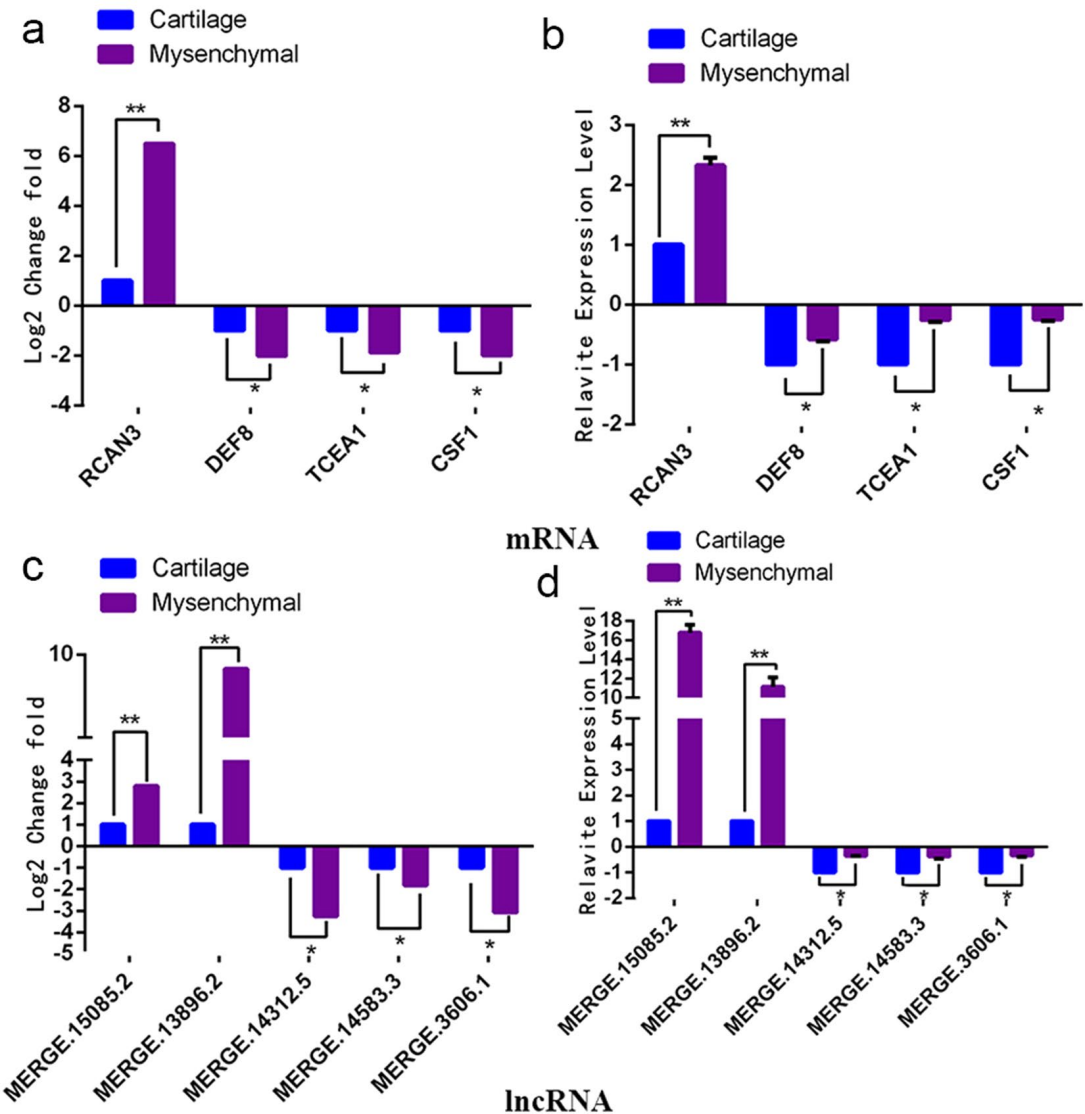
The qRT-PCR verification of lncRNAs and genes related to osteogenic differentiation. (**a**,**c**) The expression level of lncRNAs and mRNAs in high-throughput results analysis. (**b,d**) The verification results of lncRNAs and mRNAs. The data from qRT-PCR indicated that these lncRNAs and genes were differentially expressed in mesenchymal and cartilage tissue, which was consistent with the results of high-throughput sequencing.

The results of qRT-PCR further verified that these genes and lncRNAs were differentially expressed in mesenchymal tissue and cartilage tissue, which was consistent with the results of high-throughput sequencing (Fig. 5a–d).

**Figure 6:**
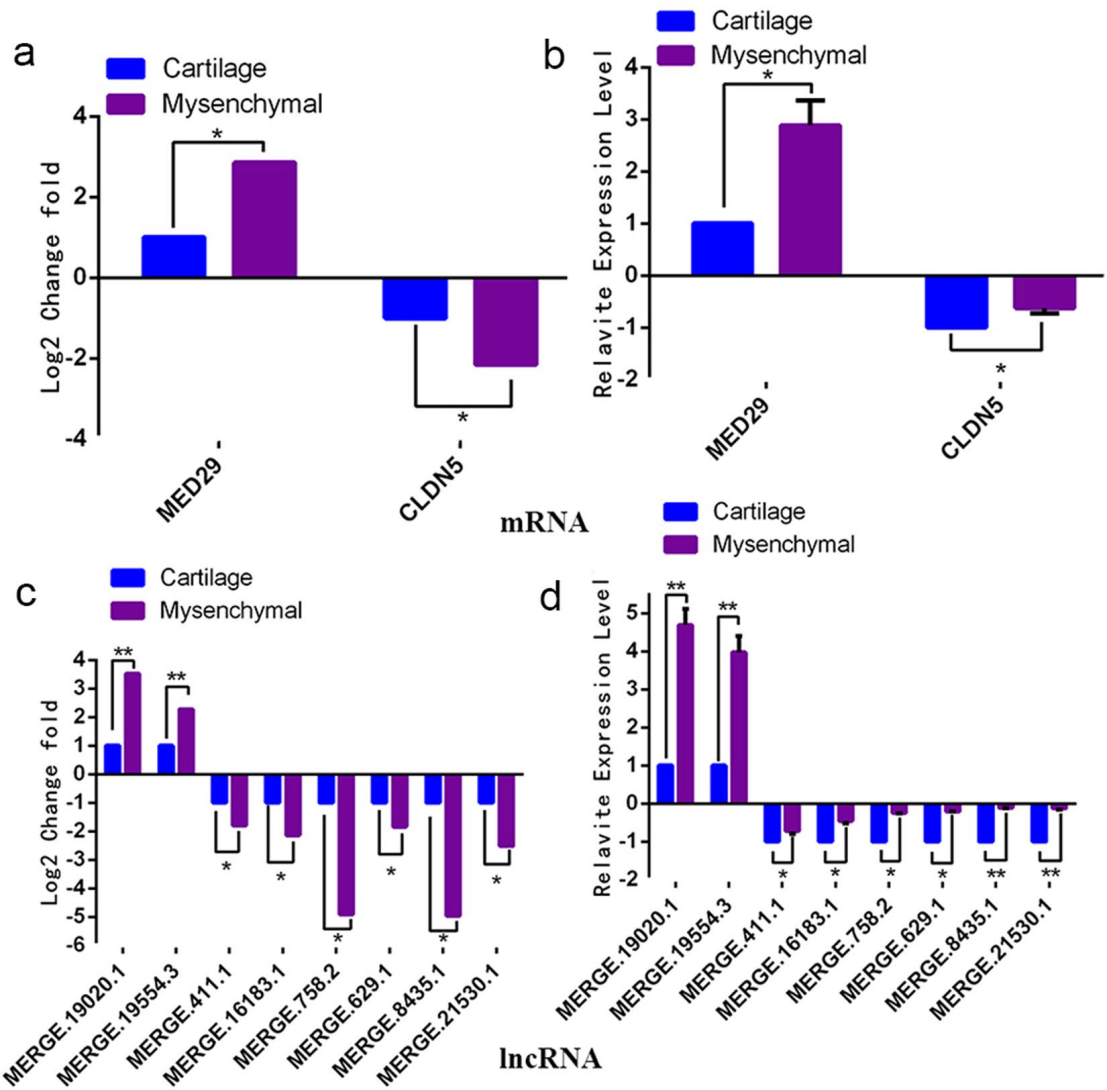
The qRT-PCR verification of lncRNAs and genes related to cell proliferation. (**a,c**) The expression level of lncRNAs and mRNAs in high-throughput results analysis. (**b**,**d)** The verification results of lncRNAs and mRNAs. These data from qRT-PCR showed that these lncRNAs and genes were differentially expressed in mesenchymal and cartilage tissue, which was consistent with the results of high-throughput sequencing.

In the regulatory network of genes related to cell proliferation, we identified two genes, MED29 (Celaphus_00004417) and CLDN5 (Celaphus_00000024), regulated by eight lncRNAs that were MERGE.16183.1, MERGE.629.1, MERGE.21530.1, MERGE.758.2, MERGE.411.1, MERGE.8435.1, MERGE. 19020.1, and MERGE. 19554.3. The qRT-PCR results further confirmed that these genes and lncRNAs were differentially expressed in mesenchymal tissue and cartilage tissue, and the difference trend was consistent with the high-throughput sequencing results (Fig. 6a–d).

**Figure 7:**
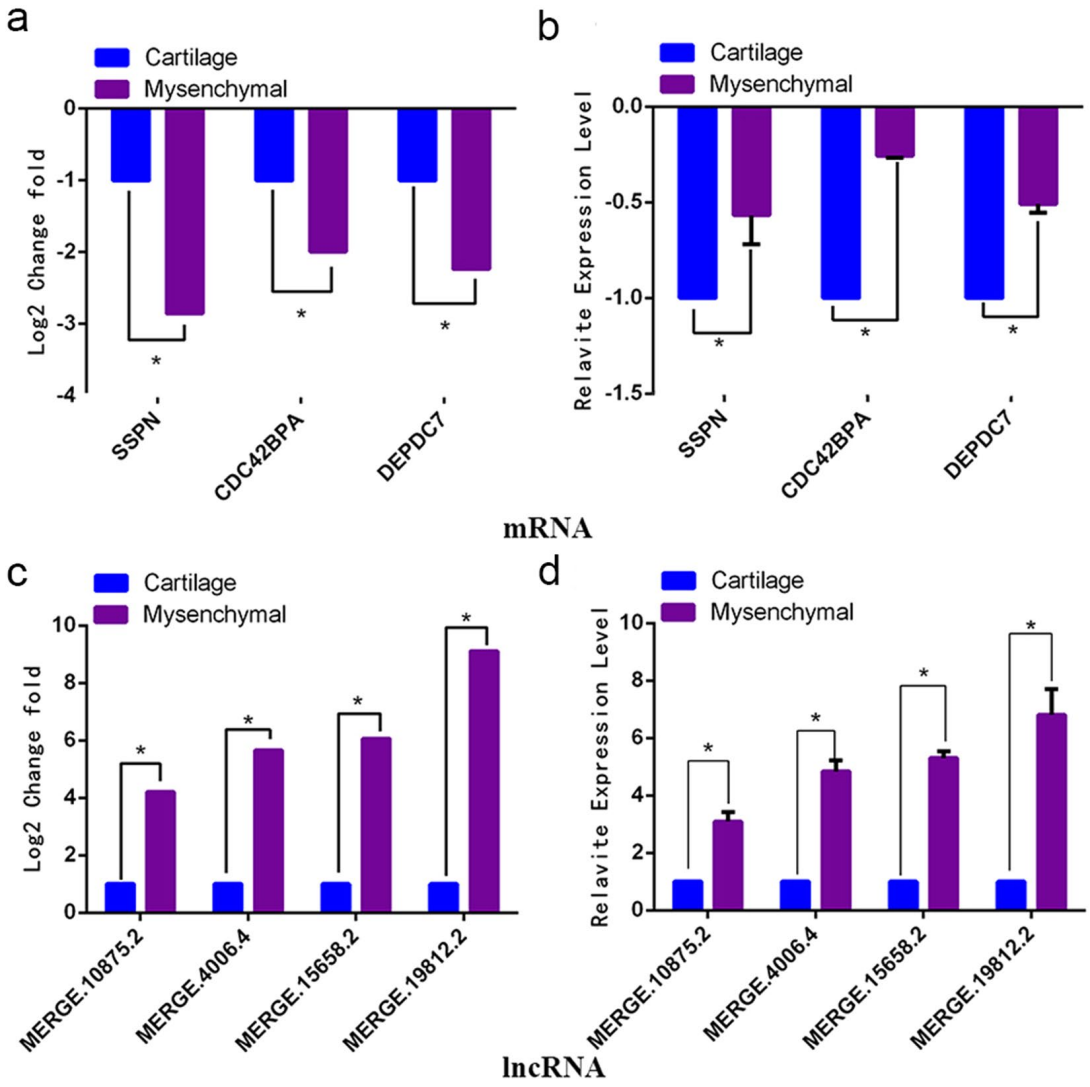
The qRT-PCR verification of lncRNAs and genes related to cell migration. (**a,c**) The expression of lncRNAs and mRNAs in high-throughput results analysis. (**b,d**) The verification results of lncRNA and mRNA. These results revealed that the lncRNAs and genes were differentially expressed in mesenchymal and cartilage tissue, which was consistent with the results of high-throughput sequencing.

Consecutively, in the regulatory network of migration-related genes, three related target genes, namely SSPN (Celaphus_00014062), CDC42BPA (Celaphus_00010867), and DEPDC7 (Celaphus_00009291) may be the targets of four lncRNAs including MERGE.10875.2, MERGE.15658.2, MERGE.4006.4, and MERGE.19812.2. The results of qRT-PCR indicated that these genes and lncRNAs were differentially expressed in mesenchymal and cartilage tissues, and these were consistent with the high-throughput results (Fig. 7a–d). Thus, it could be speculated that lncRNAs play a critical role in the osteogenic differentiation, rapid proliferation, and migration in the tip tissues of deer antler.

### Validation of specific interaction between lncRNA and mRNA

**Figure 8:**
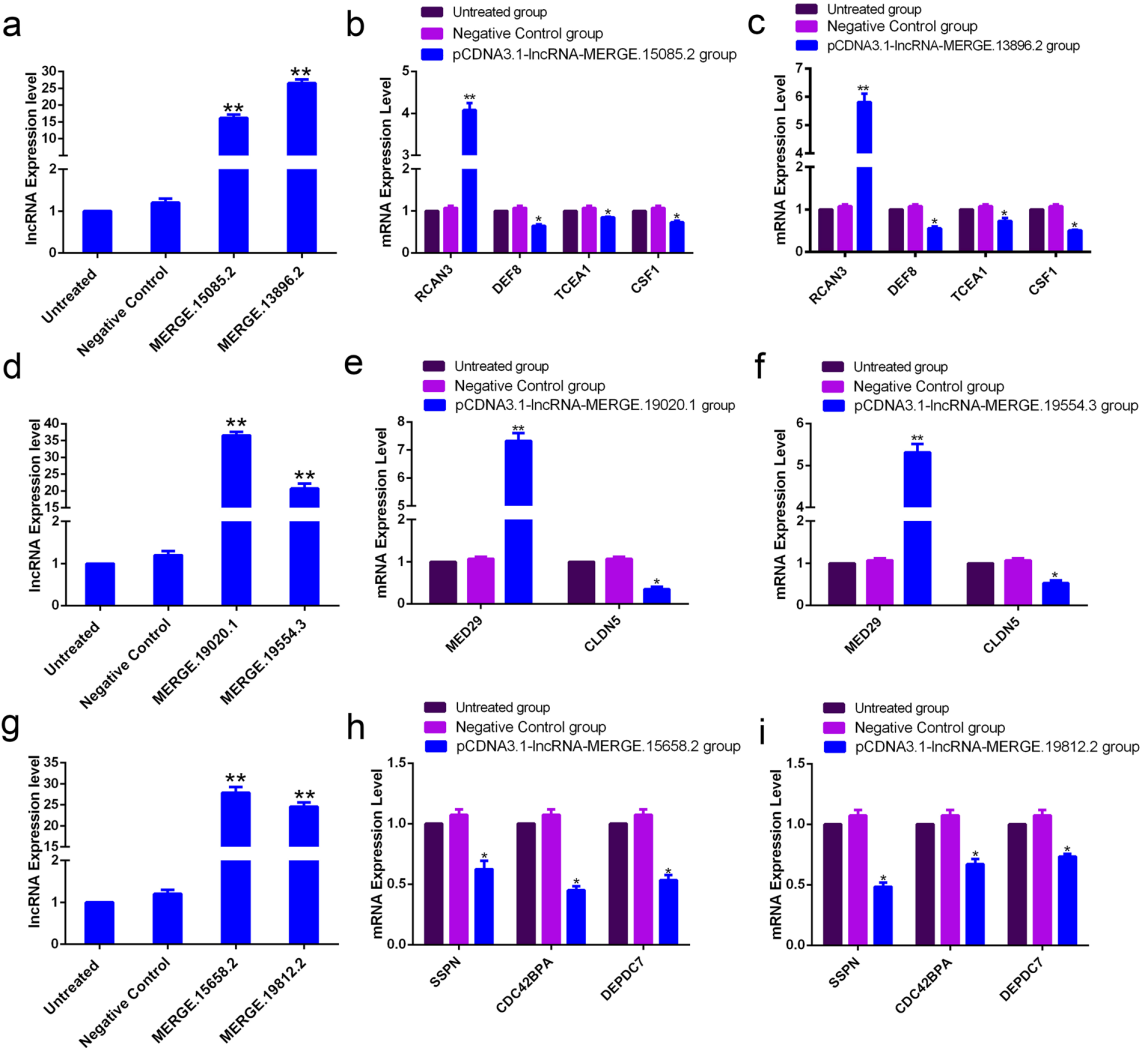
Validation of specific interaction between lncRNA and mRNA. (**a**) qRT-PCR to verify the overexpression levels of most differentially lncRNA MERGE.15085.2 and MERGE.13896.2 related to osteogenic differentiation. (**b**,**c**) The effect of MERGE.15085.2 and MERGE.13896.2 on the expression levels of genes related to osteogenic differentiation. (**d**) qRT-PCR to verify the overexpression levels of most differentially MERGE.19020.1 and MERGE.19554.3 related to cell proliferation. (**e**,**f**) lncRNA MERGE .19020.1 and MERGE.19554.3 effect on the expression levels of mRNAs related to cell proliferation. (**g**) qRT-PCR to verify the overexpression levels of most differentially lncRNA MERGE.15658.2 and MERGE.19812.2 related to cell migration. (**h**,**i**) The effect of MERGE.15658.2 and MERGE.19812.2 on the expression levels of genes related to cell migration. These results further indicated the interaction between differentially lncRNA and mRNA.

In the regulatory network of genes related to osteogenic differentiation, we verified the interactions between most significant differentially expressed lnc-MERGE.15085.2 and lnc-MERGE.13896.2 and RCAN3, DEF8, TCEA1, and CSF1 genes. qRT-PCR results showed that when lnc-MERGE.15085.2 was overexpressed, the expression level of RCAN3 mRNA was upregulated, while that of DEF8, TCEA1, and CSF1 genes was downregulated. When another lnc-MERGE.13896.2 was overexpressed, the expression level of RCAN3 mRNA was upregulated, while that of DEF8, TCEA1, and CSF1 was downregulated (Fig. 8a–c). This result further confirmed the specific interaction between the genes associated with osteogenic differentiation and two most differentially expressed lncRNAs.

In the regulatory network of genes related to cell proliferation, we selected lnc-MERGE.19020.1 and lnc-MERGE.19554.3, which are most differentially expressed, to verify the interactions with genes, MED29 and CLDN5. qRT-PCR results indicated that when lnc-MERGE.19020.1 was overexpressed, the mRNA expression level of MED29 gene increased, while that of CLDN5 gene decreased. Furthermore, when lnc-MERGE.19554.3 was overexpressed, the mRNA expression level of the MED29 gene increased, while that of the CLDN5 gene decreased (Fig. 8d–f). The correlation between the genes related to cell proliferation and the two most differentially expressed lncRNAs was verified again.

In the regulatory network of cell migration-related genes, the correlations between the most significantly differentially expressed lnc-MERGE.15658.2 and lnc-MERGE.19812.2 and the related genes SSPN, CDC42BPA, and DEPDC7 were verified. qRT-PCR results demonstrated that when two lncRNAs were overexpressed, the expressions of SSPN, CDC42BPA, and DEPDC7 genes were downregulated (Fig. 8g–i), indicating a correlation between genes related to cell migration and two most differentially expressed lncRNAs.

The verification of the lncRNA-mRNA interaction correlation revealed that these related genes might be one of the targets of these most differentially expressed lncRNAs. These results suggested that these lncRNAs not only have positive and negative regulatory effects but also prove critical regulatory effects in the osteogenic differentiation, cell proliferation, and migration of the antler tissue.

## Discussion

Antler completely regenerates every year, which is a complicated process, including various growth factors^15^. The vertical growth of deer antler is completed by the cartilage osteogenesis at the top of each branch, and the process of intraperiosteal ossification is effectuated within the periosteum. The periosteum around the antlers is connected to the perichondrium, while the dense cell layer below the perichondrium is mesenchymal. The mesenchymal cells have a strong ability to proliferate and rapidly differentiate from mesenchyme to cartilage, in order to promote the rapid deer antler growth, wherein a variety of cell growth factors are involved^16^. All growth factors can stimulate the growth and differentiation of antler mesenchyma, cartilage, osteogenesis, and epithelium. Eventually, these growth factors lead to the continued recovery and regeneration of antler^17^. Previous studies demonstrated that miRNAs regulate a variety of growth factors, and thus, play a critical role in the regulation of antler; however, the effects of lncRNAs on the regeneration mechanism of antler have been rarely reported.

Previously, lncRNAs were considered as biologically non-functional transcription “noise” Nowadays, lncRNAs have been found to be involved in various biological processes. Although lncRNA could not be translated into protein, it is crucial in the regulation of gene expression^18^. The expression patterns of different lncRNAs regulate the cell cycle, proliferation, migration, and cell differentiation. Also, it performs functions through a variety of mechanisms, such as acting as a scaffold, bait, signal, and guide^19^. He *et al*. showed an evolutionarily conserved and widespread lncRNA-taurine upregulated gene 1 (TUG1) in osteoblast-induced periodontal ligament stem cells. The induction of the expression of TUG1 and detection of the related osteogenic markers revealed that TUG1 promotes osteoblast differentiation. The knockdown of the gene inhibited the osteogenic potential of periodontal ligament stem cells. Thus, TUG1 may regulate osteoblast differentiation by regulating the expression of Lin28^20^. Liao *et al*. proposed that lncRNAH19 serves as the mediator of BMP9 signal transduction by regulating the Notch signaling pathway. lncRNAH19 may be essential for BMP9-induced osteogenic differentiation of MSCs, and the dysregulation of H19 expression may impair normal osteogenesis, leading to pathogenic processes such as bone tumor development^21^. The host gene 7 (SNHG7) of nucleolar RNA is a newly identified carcinogenic long non-coding RNA in cancer. Wang proposed that SNHG7 enhances cell proliferation and inhibits apoptosis by downregulating the expression of P15 and P16 in gastric cancer^22^. Zhang *et al*. also discovered that SNHG7 enhanced the cell migration from gastric cancer by inhibiting the miR-34a-Snail- EMT axis and invasion^23^. These studies showed that lncRNA plays a major role in osteogenesis; however, only a few reports described the role of lncRNA in antler osteogenesis.

In order to investigate the role of lncRNA in the development of antler, high-throughput sequencing was performed on mesenchymal and cartilage tissues. Thus, we considered that most lncRNAs could be distributed in the deer genome without position preference. Concurrently, high-throughput sequencing revealed a large number of differentially expressed lncRNAs and mRNAs in mesenchymal tissue and cartilage tissue, based on which, the lncRNA-mRNA network was constructed to analyze the function of lncRNAs. The reliability of the differential expression of lncRNAs and mRNAs were evaluated by qRT-PCR. In order to further study the role of lncRNAs in deer antler regeneration and differentiation, We performed GO and KEGG analysis on differentially expressed mRNA, these differentially expressed mRNAs were mainly concentrated in signaling pathways such as osteoclast differentiation, hematopoietic cell lineage, Ras signaling pathway, calcium signaling pathway, and PI3K-Akt signaling pathway. Osteoclast differentiation could stimulate the formation of osteoblasts and participate in bone resorption and formation^24,25^. The calcium signaling pathway plays a key role in regulating cell function. The process of Ca^2+^ influx into cells generates biological signals that regulate intracellular processes and play a major role in the proliferation and osteogenic differentiation of bone marrow mesenchymal stem cells^26^. Ras signaling pathway serves as a molecular switch to regulate cell proliferation, survival, growth, migration, differentiation, or signaling pathways for the regulation of cytoskeletal viability^27^. PI3K-Akt signaling pathway is involved in apoptosis, cell proliferation, growth, and differentiation^28^. Ma *et al*. pointed out that the activation of PI3K/Akt signaling pathway was essential for rat osteoblast proliferation and differentiation^29^. Lin showed that PI3K/Akt signaling enhances osteoblast proliferation and differentiation by activating the NF-κB pathway^30^. Some studies have suggested that the PI3K/Akt signaling pathway could be most relevant to the development of staghorn and participate in the proliferation of AP and PP cells and differentiation.

The above results suggested that the change in the lncRNA in antler tip tissue was important for osteogenic differentiation, cell proliferation, and migration. The genes and their corresponding lncRNA-related osteogenesis, cell proliferation, and migration were randomly selected to construct a network diagram. RCAN3, is a calcineurin that affects osteoclasts and participates in osteoclast differentiation^31^. CSF1 is one of the cytokines directly involved in osteoclast differentiation, which could actively regulate the proliferation, differentiation of monocytes, and maintain its activity. In addition, CSF1 regulates the placental function and promotes bone resorption^32^. DEF8 regulates the distribution of lysosomes, the formation of wrinkle boundaries in osteoclasts, and participates in bone resorption^33^. TCEA1 is a functional gene that regulates the expansion of myeloid cells and affects the growth, necessary for efficient RNA polymerase II transcription elongation past template-encoded arresting sites^34^. Mediator complex subunit 29 (MED29) is part of a large multiprotein coactivator complex that mediates regulatory signals from gene-specific activators to general transcription machinery in RNA polymerase II-mediated transcription, MED29 can affect the growth and differentiation of cells by participating in transcriptional regulation^35^. CLDN5 is a member of the claudin family and the main protein that forms the tight junctions between the cells^36^. SSPN enhances the diffusion of complex proteins to the recruitment on the cell surface. Gibbs *et al*. showed that the overexpression of SSPN reduces dystrophic histopathology in the diaphragm and provides a structural link between the submuscular cell bone and the extracellular matrix of muscle cells^37^. CDC42BPA has been shown to localize to the lamella of mammalian cells through interaction with an adaptor protein, leucine repeat adaptor protein 35a (LRAP35a), which links it to myosin 18 A (MYO18A) for the activation of the lamellar actomyosin network essential for cell migration^38^ and is also crucial for cell division and migration^39^. DEPDC7 participates in cell proliferation and migration. Liao *et al*. found that the overexpression of DEPDC7 in hepatocellular carcinoma cell lines inhibited the cell proliferation, growth, movement, and invasion. The knockout of DEPDC7 promotes cell growth, migration, and invasion in hepatocellular carcinoma, which indicates that it is related to the proliferation and migration of liver cancer cells^40^. Therefore, the above genes and lncRNAs may be related to osteogenesis and cell proliferation and migration.

Based on these studies, the genes related to osteogenesis, cell proliferation, and migration and their corresponding lncRNAs were verified by qRT-PCR. Both could be differentially expressed in deer antler mesenchymal and cartilage tissues. In addition, the experiments of validation of specific interaction between lncRNA and mRNA further proved that the genes related to osteogenic differentiation, cell proliferation, and migration may be the targets of these lncRNAs. These results also indicated that lncRNAs are crucial for osteogenic differentiation, rapid proliferation, and migration in the tip tissues of deer antler. Coding genes may be controlled by multiple lncRNAs, which also suggests that lncRNAs affect biological processes by influencing these genes; however, their complex regulatory mechanisms need to be investigated further.

In this study, an interaction network diagram with genes and lncRNA related to osteogenesis, proliferation, migration was constructed. As observed, the lncRNA was found to promote osteogenic differentiation and proliferation of deer antler tissue. During the rapid growth of deer antler, the tightly regulated correlation between ncRNA and mRNA may be one of the reasons for the development of antler; however, the specific mechanism is yet unclear. In conclusion, the current results provided a new theoretical basis for the reproductive development of deer antlers.

## Materials and methods

### Collection of samples

The tip tissues of three healthy male sika deer antlers with the same living conditions at 3-years-old were collected and cut off at a distance of 4–5 cm from the tip of the antler in a direction perpendicular to the growth axis. According to the method reported by Li *et al*.^41^, the mesenchymal and cartilage tissues were separated, and total RNA was extracted. Then, the RNA integrity and concentration were assessed by agarose gel electrophoresis and Agilent 2100 (260/280 instrument).

This study was approved by the Experimental Animal Ethics Committee of Jilin Agricultural University (license number ECLA-JLAU-17031). All methods were performed in accordance with the relevant guidelines and regulations.

### Library construction and sequencing

Data were filtered based on raw reading output from the Illumina Nova Seq. 6000 platform to obtain clean data. All downstream analyses were based on high-quality cleaning data. Finally, software HISAT2 2.1.0^42^ was used to map the sample data to the reference genome, and the reference genome was obtained from https://www.ncbi.nlm.nih.gov/genome/?term=Cervus.

### Identification of lncRNA

According to the lncRNA length threshold, transcript length filtering was performed to remove transcripts shorter than 200 bp. Then, the filter of fragments per kilobases per million fragments (FPKM) was applied. For assembly transcripts with only one exon, transcripts with FPKM ≥ 2 were retained, and for assembly transcripts with multiple exons, transcripts with FPKM ≥ 0.5 were retained. The comparison with the reference genome may yield known lncRNAs. For the assembled unknown transcripts, Stringtie 1.3.3^43^ software was used to filter the encoding ability, and then the CPC 0.9r2^44^, CNCI^45^, and HMMER 3.1b1^46^ software were utilized to predict the encoding ability. When all three software predicted no coding ability, those were termed as novel lncRNAs.

### Identification and analysis of differentially expressed lncRNAs and mRNAs

The DESeq. 1.28.0^47^ software was used to normalize and estimate the variance of the cleaning data in mesenchymal tissue and cartilage tissue of each group, followed by the analysis of differential expression. The criteria of screening for the differences in the lncRNA and mRNA expression were | log_2_FC| ≥ 1 and P-value ≤ 0.05.

### Co-expression network of mRNA-lncRNA

Most lncRNAs are unannotated, and their function is unknown. In order to predict the function of these lncRNAs, the correlation between the expression values of all samples of differential lncRNA and mRNA was evaluated. The default filtering criteria was P < 0.05, and the expression correlation coefficient was >0.95. Correlation test function in the R data analysis tool was used to screen out significantly related lncRNA-mRNA pairs, which were then imported into Cytoscape 6.1 software to construct the relevant network diagram. Finally, MCL v2.0 software was used to excavate the sub-modules of the total network, and genes related to osteogenesis, cell proliferation, and migration were identified by module analysis.

### Gene enrichment analysis

KOBAS 3.0^48^ was used for Gene Ontology (GO) enrichment^49^ and Kyoto Encyclopedia of Genes and Genomes (KEGG: www.kegg.jp/kegg/kegg1.html) pathway enrichment analysis^50–53^. The enrichment of GO terms (false discovery rate (FDR) < 0.05) and KEGG pathway (P < 0.05).

### Verification of lncRNA and target genes differentially expressed by qRT- PCR

Total RNA was extracted from mesenchymal and cartilage tissues and reversed transcribed into cDNA by using PrimeScript RT Reagent Kit with gDNA Eraser (TaKaRa). The qRT-PCR reaction was performed using SYBR Premix Ex Taq (TaKaRa) in a 20 μL reaction volume, including 10 μL SYBR Green Master Mix, 0.4 μL forward Primer (10 μM), 0.4 μL reverse primer (10 μM), 0.1 μL ROX, 2.0 μL cDNA, and 7.1 μL nuclease-free water. The reaction was as follows: denaturation at 95 °C for 30 s, then annealing at 95 °C for 5 s, 60 °C for 30 s, 72 °C for 30 s, for a total of 40 cycles, finally extension at 95 °C for 15 s, 60 °C for 1 min, 95 °C for 15 s The experiment was performed in triplicates, and the *β-actin* gene was used as a reference. The expression level of relative lncRNA and mRNA was calculated using the 2^−ΔΔCt^ method. T-test was applied for statistical analysis. P < 0.05 indicated statistically significant difference. Primer sequences are shown in Supplementary Table S3a.

### Validation of specific interaction between lncRNA and mRNA

In each of the regulatory networks related to osteogenic differentiation, cell proliferation, and migration, we selected two lncRNAs with the highest differences in the expression and verified their interactions with the corresponding genes. Based on high-throughput sequencing results, the full-length sequence of the screened lncRNA was synthesized, and the overexpression recombinant plasmid pCDNA3.1-lncRNA was constructed. Then, the recombinant plasmid and negative plasmid were transfected into antler chondrocytes respectively, and the cells of each group were collected after 48 h of culture. Total RNA was extracted from each group of cells, and cDNA was synthesized by reverse transcription. Finally, qRT-PCR was performed to detect the mRNA expression of related genes. The qRT-PCR reaction system and reaction conditions were the same as that of 4.7. The expression level of the target mRNA was calculated using the 2^−ΔΔCt^ method.

### Statistical analysis

The experimental data were presented as means ± standard deviation (SD). SPSS 22.0 software (IBM, Armonk, NY, USA) was used to perform all the statistical analyses. The significant differences between the groups were evaluated by Student’s t-test. The P-values <0.05 were considered statistically significant.

### Ethics statement

We confirm that all methods were carried out in accordance with relevant guidelines and regulations. This study was approved by the Experimental Animal Ethics Committee of Jilin Agricultural University (license number ECLA-JLAU-17031).

## Supplementary information

Supplementary information 1.

Supplementary information 2.

Supplementary information 3.

## Data Availability

The datasets used and/or analyzed in this study are available from the corresponding author on reasonable request.
