# Supplementary material for: Screening and functional identification of lncRNAs in antler mesenchymal and cartilage tissues using high-throughput sequencing
Source: Sci Rep. 2020 Jun 11;10:9492. doi: 10.1038/s41598-020-66383-1 (PMC7289821; doi:10.1038/s41598-020-66383-1)
Supplement: Supplementary file 1 — Supplementary information 1. [file 41598_2020_66383_MOESM1_ESM.pdf]

**Screening and functional identification of lncRNAs in antler mesenchymal and cartilage tissues using high-throughput sequencing**

Dan-yang Chen, Ren-feng Jiang, Yan-jun Li, Ming-xiao Liu, Lei Wu*, Wei Hu*

*College of Life Science, Jilin Agriculture University, Changchun, Jilin Province, 130118, China*

*Corresponding Author: Wei Hu, Lei Wu College of Life Science, Jilin Agriculture University, Changchun, Jilin Province, 130118, China.

Dan-yang Chen: cdyjlau@163.com.

Ren-feng Jiang: 437349926@qq.com.

Yan-jun Li: 1781521041@qq.com.

Ming-xiao Liu: 1361545695@qq.com.

Lei Wu: 837660996@qq.com.

Wei Hu: huweilab@126.com, Tel: +86-15699561990, Fax: +86-21-64085875

**Supplementary Table S1a.** List of Gene Ontology enrichment.

| Term | Database | ID | P-Value |
|---|---|---|---|
| extracellular matrix | Cellular Component | GO:0031012 | 1.19E-17 |
| extracellular region part | Cellular Component | GO:0044421 | 1.57E-12 |
| proteinaceous extracellular matrix | Cellular Component | GO:0005578 | 8.01E-11 |
| extracellular region | Cellular Component | GO:0005576 | 2.43E-10 |
| membrane | Cellular Component | GO:0016020 | 2.30E-08 |
| membrane part | Cellular Component | GO:0044425 | 5.33E-08 |
| plasma membrane | Cellular Component | GO:0005886 | 6.86E-08 |
| cell periphery | Cellular Component | GO:0071944 | 7.91E-08 |
| receptor complex | Cellular Component | GO:0043235 | 1.06E-07 |
| intrinsic component of membrane | Cellular Component | GO:0031224 | 1.25E-07 |
| integral component of membrane | Cellular Component | GO:0016021 | 4.68E-07 |
| intrinsic component of plasma membrane | Cellular Component | GO:0031226 | 5.82E-07 |
| integrin complex | Cellular Component | GO:0008305 | 2.82E-06 |
| protein complex involved in cell adhesion | Cellular Component | GO:0098636 | 2.82E-06 |
| plasma membrane part | Cellular Component | GO:0044459 | 2.87E-06 |
| extracellular space | Cellular Component | GO:0005615 | 4.02E-06 |
| integral component of plasma membrane | Cellular Component | GO:0005887 | 6.03E-06 |
| membrane-bounded vesicle | Cellular Component | GO:0031988 | 9.78E-06 |
| extracellular exosome | Cellular Component | GO:0070062 | 1.33E-05 |
| extracellular membrane-bounded organelle | Cellular Component | GO:0065010 | 1.33E-05 |
| system development | Biological Process | GO:0048731 | 5.64E-14 |
| single-multicellular organism process | Biological Process | GO:0044707 | 9.59E-14 |
| multicellular organismal development | Biological Process | GO:0007275 | 1.85E-13 |
| biological adhesion | Biological Process | GO:0022610 | 4.03E-13 |
| cell adhesion | Biological Process | GO:0007155 | 6.52E-13 |
| multicellular organismal process | Biological Process | GO:0032501 | 3.03E-12 |
| single-organism developmental process | Biological Process | GO:0044767 | 1.10E-11 |
| developmental process | Biological Process | GO:0032502 | 2.08E-11 |
| regulation of response to stimulus | Biological Process | GO:0048583 | 4.57E-11 |
| immune system process | Biological Process | GO:0002376 | 2.26E-10 |

| | | | |
|---|---|---|---|
| organ development | Biological Process | GO:0048513 | 4.82E-10 |
| intracellular signal transduction | Biological Process | GO:0035556 | 5.42E-10 |
| cell activation | Biological Process | GO:0001775 | 6.01E-10 |
| regulation of multicellular organismal process | Biological Process | GO:0051239 | 1.64E-09 |
| positive regulation of multicellular organismal process | Biological Process | GO:0051240 | 2.06E-09 |
| cell migration | Biological Process | GO:0016477 | 2.62E-09 |
| leukocyte differentiation | Biological Process | GO:0002521 | 2.79E-09 |
| leukocyte activation | Biological Process | GO:0045321 | 4.68E-09 |
| lymphocyte activation | Biological Process | GO:0046649 | 6.69E-09 |
| receptor activity | Molecular Function | GO:0004872 | 3.50E-09 |
| molecular transducer activity | Molecular Function | GO:0060089 | 8.37E-09 |
| guanyl-nucleotide exchange factor activity | Molecular Function | GO:0005085 | 8.25E-07 |
| signaling receptor activity | Molecular Function | GO:0038023 | 3.06E-06 |
| transmembrane receptor protein kinase activity | Moleczular Function | GO:0019199 | 4.34E-06 |
| signal transducer activity | Molecular Function | GO:0004871 | 4.83E-06 |
| transmembrane receptor protein tyrosine kinase activity | Molecular Function | GO:0004714 | 5.06E-06 |
| Ras guanyl-nucleotide exchange factor activity | Molecular Function | GO:0005088 | 7.71E-06 |
| protein tyrosine kinase activity | Molecular Function | GO:0004713 | 1.07E-05 |
| calcium ion binding | Molecular Function | GO:0005509 | 5.67E-05 |
| molecular function regulator | Molecular Function | GO:0098772 | 6.64E-05 |
| transmembrane signaling receptor activity | Molecular Function | GO:0004888 | 6.65E-05 |
| proteoglycan binding | Molecular Function | GO:0043394 | 8.36E-05 |
| fibroblast growth factor-activated receptor activity | Molecular Function | GO:0005007 | 1.44E-04 |
| cytokine receptor activity | Molecular Function | GO:0004896 | 2.21E-04 |
| very-low-density lipoprotein particle receptor activity | Molecular Function | GO:0030229 | 3.53E-04 |
| glycosaminoglycan binding | Molecular Function | GO:0005539 | 4.43E-04 |
| kinase activity | Molecular Function | GO:0016301 | 6.81E-04 |
| phospholipid binding | Molecular Function | GO:0005543 | 7.02E-04 |
| cargo receptor activity | Molecular Function | GO:0038024 | 1.22E-03 |

**Supplementary Table S1b.** List of KEGG pathway

| Term | Database | ID | P-Value |
|---|---|---|---|
| Axon guidance | KEGG PATHWAY | ko04360 | 1.74E-06 |
| Osteoclast differentiation | KEGG PATHWAY | ko04380 | 1.78E-06 |
| Lysosome | KEGG PATHWAY | ko04142 | 4.11E-06 |
| ECM-receptor interaction | KEGG PATHWAY | ko04512 | 5.27E-06 |
| Ras signaling pathway | KEGG PATHWAY | ko04014 | 8.05E-05 |
| Steroid biosynthesis | KEGG PATHWAY | ko00100 | 0.000230258 |
| Pathways in cancer | KEGG PATHWAY | ko05200 | 0.000288509 |
| Hematopoietic cell lineage | KEGG PATHWAY | ko04640 | 0.000326823 |
| Complement and coagulation cascades | KEGG PATHWAY | ko04610 | 0.000395246 |
| Central carbon metabolism in cancer | KEGG PATHWAY | ko05230 | 0.000396467 |
| Calcium signaling pathway | KEGG PATHWAY | ko04020 | 0.00045477 |
| PI3K-Akt signaling pathway | KEGG PATHWAY | ko04151 | 0.000474926 |
| Fatty acid metabolism | KEGG PATHWAY | ko01212 | 0.000514111 |
| Prostate cancer | KEGG PATHWAY | ko05215 | 0.000858631 |
| B cell receptor signaling pathway | KEGG PATHWAY | ko04662 | 0.001288316 |
| Epithelial cell signaling in Helicobacter pylori infection | KEGG PATHWAY | ko05120 | 0.002696511 |
| Inflammatory mediator regulation of TRP channels | KEGG PATHWAY | ko04750 | 0.003658155 |
| HTLV-I infection | KEGG PATHWAY | ko05166 | 0.004008901 |
| PPAR signaling pathway | KEGG PATHWAY | ko03320 | 0.004702208 |
| Fc gamma R-mediated phagocytosis | KEGG PATHWAY | ko04666 | 0.00500198 |
