# Supplementary material for: Screening and functional identification of lncRNAs in antler mesenchymal and cartilage tissues using high-throughput sequencing
Source: Sci Rep. 2020 Jun 11;10:9492. doi: 10.1038/s41598-020-66383-1 (PMC7289821; doi:10.1038/s41598-020-66383-1)
Supplement: Supplementary file 2 — Supplementary information 2. [file 41598_2020_66383_MOESM2_ESM.pdf]

Screening and functional identification of lncRNAs in antler mesenchymal and cartilage tissues using high-throughput sequencing

Dan-yang Chen, Ren-feng Jiang, Yan-jun Li, Ming-xiao Liu, Lei Wu*, Wei Hu*

College of Life Science, Jilin Agriculture University, Changchun, Jilin Province, 130118, China

*Corresponding Author: Wei Hu, Lei Wu College of Life Science, Jilin Agriculture University, Changchun, Jilin Province, 130118, China.

Dan-yang Chen: cdyjlau@163.com.

Ren-feng Jiang: 437349926@qq.com.

Yan-jun Li: 1781521041@qq.com.

Ming-xiao Liu: 1361545695@qq.com.

Lei Wu: 837660996@qq.com.

Wei Hu: huweilab@126.com, Tel: +86-15699561990, Fax: +86-21-64085875

Supplementary Table S2a. List of osteogenic differentiation-related lncRNAs.

lncRNA	Gene	Gene name	Type
MERGE.15085.2	Celaphus_00014788	RCAN3	Positive
MERGE.13896.2	Celaphus_00014788	RCAN3	Positive
MERGE.14312.5	Celaphus_00014788	RCAN3	Negative
MERGE.14583.3	Celaphus_00014788	RCAN3	Negative
MERGE.3606.1	Celaphus_00014788	RCAN3	Positive
MERGE.15085.2	Celaphus_00004075	DEF8	Negative
MERGE.13896.2	Celaphus_00004075	DEF8	Negative
MERGE.14312.5	Celaphus_00004075	DEF8	Positive
MERGE.14583.3	Celaphus_00004075	DEF8	Positive
MERGE.3606.1	Celaphus_00004075	DEF8	Positive
MERGE.15085.2	Celaphus_00016251	TCEA1	Negative
MERGE.13896.2	Celaphus_00016251	TCEA1	Negative
MERGE.14312.5	Celaphus_00016251	TCEA1	Positive
MERGE.14583.3	Celaphus_00016251	TCEA1	Positive
MERGE.3606.1	Celaphus_00016251	TCEA1	Positive
MERGE.15085.2	Celaphus_00002219	CSF1	Negative
MERGE.13896.2	Celaphus_00002219	CSF1	Negative
MERGE.14312.5	Celaphus_00002219	CSF1	Positive
MERGE.14583.3	Celaphus_00002219	CSF1	Negative
MERGE.3606.1	Celaphus_00002219	CSF1	Positive
MERGE.11390.2	Celaphus_00016251	TCEA1	Positive
MERGE.346.6	Celaphus_00016251	TCEA1	Positive
MERGE.4296.2	Celaphus_00016251	TCEA1	Positive
MERGE.2925.1	Celaphus_00000653	TBX2	Positive
MERGE.9033.3	Celaphus_00000653	TBX2	Positive
MERGE.13896.2	Celaphus_00000653	TBX2	Positive
MERGE.4296.2	Celaphus_00002219	CSF1	Positive
MERGE.11390.2	Celaphus_00004075	DEF8	Positive
MERGE.346.6	Celaphus_00004075	DEF8	Positive
MERGE.346.6	Celaphus_00003694	hypothetical protein	Positive
MERGE.2925.1	Celaphus_00014788	RCAN3	Positive
MERGE.9033.3	Celaphus_00014788	RCAN3	Positive
MERGE.22085.2	Celaphus_00014788	RCAN3	Positive
MERGE.3606.1	Celaphus_00017037	PTCH1	Positive
MERGE.3606.1	Celaphus_00000126	SEC14L4	Positive
MERGE.3606.1	Celaphus_00000126	SEC14L4	Positive
MERGE.2925.1	Celaphus_00016251	TCEA1	Positive
MERGE.9033.3	Celaphus_00016251	TCEA1	Negative
MERGE.22085.2	Celaphus_00016251	TCEA1	Negative
MERGE.11390.2	Celaphus_00000653	TBX2	Negative
MERGE.346.6	Celaphus_00000653	TBX2	Negative

MERGE.3606.1	Celaphus_00000653	TBX2	Negative
MERGE.3606.1	Celaphus_00000653	TBX2	Negative
MERGE.3606.1	Celaphus_00000653	TBX2	Negative
MERGE.2925.1	Celaphus_00002219	CSF1	Negative
MERGE.22085.2	Celaphus_00002219	CSF1	Negative
MERGE.2925.1	Celaphus_00004075	DEF8	Negative
MERGE.9033.3	Celaphus_00004075	DEF8	Negative
MERGE.22085.2	Celaphus_00004075	DEF8	Negative
MERGE.7471.3	Celaphus_00003694	hypothetical protein	Negative
MERGE.11390.2	Celaphus_00014788	hypothetical protein	Negative
MERGE.346.6	Celaphus_00014788	hypothetical protein	Negative

Supplementary Table S2b. List of cell proliferation-related lncRNAs.

lncRNA	Gene	Gene name	Type
MERGE.19020.1	Celaphus_00004417	MED29	Positive
MERGE.19554.3	Celaphus_00004417	MED29	Positive
MERGE.411.1	Celaphus_00004417	MED29	Negative
MERGE.16183.1	Celaphus_00004417	MED29	Negative
MERGE.758.2	Celaphus_00004417	MED29	Negative
MERGE.629.1	Celaphus_00004417	MED29	Negative
MERGE.8435.1	Celaphus_00004417	MED29	Negative
MERGE.21530.1	Celaphus_00004417	MED29	Negative
MERGE.19020.1	Celaphus_00000024	CLDN5	Negative
MERGE.19554.3	Celaphus_00000024	CLDN5	Negative
MERGE.411.1	Celaphus_00000024	CLDN5	Positive
MERGE.16183.1	Celaphus_00000024	CLDN5	Positive
MERGE.758.2	Celaphus_00000024	CLDN5	Positive
MERGE.629.1	Celaphus_00000024	CLDN5	Positive
MERGE.8435.1	Celaphus_00000024	CLDN5	Positive
MERGE.21530.1	Celaphus_00000024	CLDN5	Positive
MERGE.1556.4	Celaphus_00006623	ERO1A	Positive
MERGE.5681.1	Celaphus_00006623	ERO1A	Positive
MERGE.7952.1	Celaphus_00006623	ERO1A	Positive
MERGE.14209.1	Celaphus_00009543	hypothetical protein	Positive
MERGE.6168.5	Celaphus_00009543	hypothetical protein	Positive
MERGE.9208.9	Celaphus_00009543	hypothetical protein	Positive
MERGE.16183.1	Celaphus_00009543	hypothetical protein	Positive
MERGE.629.1	Celaphus_00009543	hypothetical protein	Positive
MERGE.21530.1	Celaphus_00009543	hypothetical protein	Positive
MERGE.758.2	Celaphus_00009543	hypothetical protein	Positive
MERGE.411.1	Celaphus_00009543	hypothetical protein	Positive

MERGE.8435.1	Celaphus_00009543	hypothetical protein	Positive
MERGE.7893.1	Celaphus_00000024	CLDN5	Positive
MERGE.5570.1	Celaphus_00000024	CLDN5	Positive
MERGE.5681.1	Celaphus_00004417	MED29	Positive
MERGE.5685.3	Celaphus_00004417	MED29	Positive
MERGE.5829.1	Celaphus_00004229	hypothetical protein	Positive
MERGE.16183.1	Celaphus_00004229	hypothetical protein	Positive
MERGE.758.2	Celaphus_00004229	hypothetical protein	Positive
MERGE.411.1	Celaphus_00004229	hypothetical protein	Positive
MERGE.21829.1	Celaphus_00011654	METTL1	Positive
MERGE.5829.1	Celaphus_00006623	ERO1A	Negative
MERGE.6168.5	Celaphus_00006623	ERO1A	Negative
MERGE.758.2	Celaphus_00006623	ERO1A	Negative
MERGE.19020.1	Celaphus_00009543	hypothetical protein	Negative
MERGE.19554.3	Celaphus_00009543	hypothetical protein	Negative
MERGE.5685.3	Celaphus_00009543	hypothetical protein	Negative
MERGE.21829.1	Celaphus_00000024	CLDN5	Negative
MERGE.5829.1	Celaphus_00004417	MED29	Negative
MERGE.6168.5	Celaphus_00004417	MED29	Negative
MERGE.1760.1	Celaphus_00004417	MED29	Negative
MERGE.19020.1	Celaphus_00004229	hypothetical protein	Negative
MERGE.19554.3	Celaphus_00004229	hypothetical protein	Negative
MERGE.9208.9	Celaphus_00011654	METTL1	Negative
MERGE.21530.1	Celaphus_00011654	METTL1	Negative
MERGE.7893.1	Celaphus_00011654	METTL1	Negative
MERGE.5570.1	Celaphus_00011654	METTL1	Negative

Supplementary Table S2c. List of migration-related lncRNAs.

lncRNA	Gene	Gene name	Type
MERGE.10875.2	Celaphus_00014062	SSPN	Negative
MERGE.4006.4	Celaphus_00014062	SSPN	Negative
MERGE.15658.2	Celaphus_00014062	SSPN	Negative
MERGE.19812.2	Celaphus_00014062	SSPN	Negative
MERGE.10875.2	Celaphus_00010867	CDC42BPA	Negative
MERGE.4006.4	Celaphus_00010867	CDC42BPA	Negative
MERGE.15658.2	Celaphus_00010867	CDC42BPA	Negative
MERGE.19812.2	Celaphus_00010867	CDC42BPA	Negative
MERGE.10875.2	Celaphus_00009291	DEPDC7	Negative
MERGE.4006.4	Celaphus_00009291	DEPDC7	Negative
MERGE.15658.2	Celaphus_00009291	DEPDC7	Negative
MERGE.19812.2	Celaphus_00009291	DEPDC7	Negative
MERGE.85.3	Celaphus_00008041	NEURL2	Positive
MERGE.7421.1	Celaphus_00008041	NEURL2	Positive
MERGE.3103.4	Celaphus_00008041	NEURL2	Positive
MERGE.20347.2	Celaphus_00014062	SSPN	Positive

MERGE.85.3	Celaphus_00014062	SSPN	Positive
MERGE.7421.1	Celaphus_00014062	SSPN	Positive
MERGE.16580.2	Celaphus_00014062	SSPN	Positive
MERGE.1293.2	Celaphus_00014062	SSPN	Positive
MERGE.4601.1	Celaphus_00014062	SSPN	Positive
MERGE.3103.4	Celaphus_00014062	SSPN	Positive
MERGE.399.1	Celaphus_00014062	SSPN	Positive
MERGE.20347.2	Celaphus_00010867	CDC42BPA	Positive
MERGE.4601.1	Celaphus_00010867	CDC42BPA	Positive
MERGE.3103.4	Celaphus_00010867	CDC42BPA	Positive
MERGE.85.3	Celaphus_00008338	PLAC9	Positive
MERGE.7421.1	Celaphus_00008338	PLAC9	Positive
MERGE.16580.2	Celaphus_00008338	PLAC9	Positive
MERGE.3103.4	Celaphus_00008338	PLAC9	Positive
MERGE.11572.1	Celaphus_00008338	PLAC9	Positive
MERGE.10875.2	Celaphus_00017890	hypothetical protein	Positive
MERGE.3762.4	Celaphus_00017890	hypothetical protein	Positive
MERGE.16528.4	Celaphus_00017890	hypothetical protein	Positive
MERGE.15658.2	Celaphus_00017890	hypothetical protein	Positive
MERGE.5715.5	Celaphus_00017890	hypothetical protein	Positive
MERGE.4006.4	Celaphus_00017890	hypothetical protein	Positive
MERGE.19812.2	Celaphus_00017890	hypothetical protein	Positive
MERGE.11061.3	Celaphus_00017890	hypothetical protein	Positive
MERGE.20238.4	Celaphus_00017890	hypothetical protein	Positive
MERGE.4017.2	Celaphus_00017890	hypothetical protein	Positive
MERGE.85.3	Celaphus_00009291	DEPDC7	Positive
MERGE.7421.1	Celaphus_00009291	DEPDC7	Positive
MERGE.4601.1	Celaphus_00009291	DEPDC7	Positive
MERGE.399.1	Celaphus_00009291	DEPDC7	Positive
MERGE.14728.1	Celaphus_00009294	HIPK3	Positive
MERGE.4601.1	Celaphus_00009294	HIPK3	Positive
MERGE.85.3	Celaphus_00002608	hypothetical protein	Positive
MERGE.7421.1	Celaphus_00002608	hypothetical protein	Positive
MERGE.16580.2	Celaphus_00002608	hypothetical protein	Positive
MERGE.3103.4	Celaphus_00002608	hypothetical protein	Positive
MERGE.11572.1	Celaphus_00002608	hypothetical protein	Positive
MERGE.20347.2	Celaphus_00018274	hypothetical protein	Positive
MERGE.4980.1	Celaphus_00018274	hypothetical protein	Positive
MERGE.4601.1	Celaphus_00018274	hypothetical protein	Positive
MERGE.21644.2	Celaphus_00008041	NEURL2	Negative
MERGE.15658.2	Celaphus_00008041	NEURL2	Negative
MERGE.20238.4	Celaphus_00008041	NEURL2	Negative
MERGE.4017.2	Celaphus_00008041	NEURL2	Negative

MERGE.16528.4	Celaphus_00014062	SSPN	Negative
MERGE.5715.5	Celaphus_00014062	SSPN	Negative
MERGE.11061.3	Celaphus_00014062	SSPN	Negative
MERGE.20238.4	Celaphus_00014062	SSPN	Negative
MERGE.4017.2	Celaphus_00014062	SSPN	Negative
MERGE.3762.4	Celaphus_00010867	CDC42BPA	Negative
MERGE.16528.4	Celaphus_00010867	CDC42BPA	Negative
MERGE.5715.5	Celaphus_00010867	CDC42BPA	Negative
MERGE.11061.3	Celaphus_00010867	CDC42BPA	Negative
MERGE.20347.2	Celaphus_00007437	hypothetical protein	Negative
MERGE.1293.2	Celaphus_00007437	hypothetical protein	Negative
MERGE.399.1	Celaphus_00007437	hypothetical protein	Negative
MERGE.21644.2	Celaphus_00008338	PLAC9	Negative
MERGE.15658.2	Celaphus_00008338	PLAC9	Negative
MERGE.20238.4	Celaphus_00008338	PLAC9	Negative
MERGE.4017.2	Celaphus_00008338	PLAC9	Negative
MERGE.20347.2	Celaphus_00017890	hypothetical protein	Negative
MERGE.4601.1	Celaphus_00017890	hypothetical protein	Negative
MERGE.3103.4	Celaphus_00017890	hypothetical protein	Negative
MERGE.4017.2	Celaphus_00009291	DEPDC7	Negative
MERGE.3762.4	Celaphus_00009294	HIPK3	Negative
MERGE.15658.2	Celaphus_00009294	HIPK3	Negative
MERGE.4006.4	Celaphus_00009294	HIPK3	Negative
MERGE.19812.2	Celaphus_00009294	HIPK3	Negative
MERGE.21644.2	Celaphus_00002608	hypothetical protein	Negative
MERGE.16528.4	Celaphus_00002608	hypothetical protein	Negative
MERGE.15658.2	Celaphus_00002608	hypothetical protein	Negative
MERGE.11061.3	Celaphus_00002608	hypothetical protein	Negative
MERGE.20238.4	Celaphus_00002608	hypothetical protein	Negative
MERGE.4017.2	Celaphus_00002608	hypothetical protein	Negative
MERGE.4006.4	Celaphus_00018274	hypothetical protein	Negative
MERGE.19812.2	Celaphus_00018274	hypothetical protein	Negative
