# Supplementary material for: Screening and functional identification of lncRNAs in antler mesenchymal and cartilage tissues using high-throughput sequencing
Source: Sci Rep. 2020 Jun 11;10:9492. doi: 10.1038/s41598-020-66383-1 (PMC7289821; doi:10.1038/s41598-020-66383-1)
Supplement: Supplementary file 3 — Supplementary information 3. [file 41598_2020_66383_MOESM3_ESM.pdf]

Screening and functional identification of lncRNAs in antler mesenchymal and cartilage tissues using high-throughput sequencing

Dan-yang Chen, Ren-feng Jiang, Yan-jun Li, Ming-xiao Liu, Lei Wu*, Wei Hu*

College of Life Science, Jilin Agriculture University, Changchun, Jilin Province, 130118, China

*Corresponding Author: Wei Hu, Lei Wu College of Life Science, Jilin Agriculture University, Changchun, Jilin Province, 130118, China.

Dan-yang Chen: cdyjlau@163.com.

Ren-feng Jiang: 437349926@qq.com.

Yan-jun Li: 1781521041@qq.com.

Ming-xiao Liu: 1361545695@qq.com.

Lei Wu: 837660996@qq.com.

Wei Hu: huweilab@126.com, Tel: +86-15699561990, Fax: +86-21-64085875

Supplementary Table S3a. The primer sequences of qRT-PCR.

Primer	Sequence(5′–3′)
MERGE.3606.1-R	GGGCGCATGGGTTTGTT
MERGE.3606.1-F	CGTGGAAGTGTTCACGGGA
MERGE.15085.2-R	CTCGCCACGTCTCGAAAAC
MERGE.15085.2-F	GCACACTCCTGGTCCCAACTA
MERGE.14312.5-R	GCCTGTCTTTGGGAGATTCG
MERGE.14312.5-F	CTTTTGGATGATGACGCAGC
MERGE.14583.3-R	GCAGTGAAACCTTGCAGCG
MERGE.14583.3-F	GGTATGGGGGAAGTGGAACA
MERGE.13896.2-R	TCGGAGAAATTGCGGGC
MERGE.13896.2-F	CGGTGCTCAAAGTCGGTGT
(Celaphus_00002219) CSF1-R	GCCAGCAAGACCAGGATGATA
(Celaphus_000022190 CSF1-F	GAGAGGCAGCACAAGGAAGC
(Celaphus_00004075) DEF8-R	AGCTGCCAGAACACTCGGA
(Celaphus_00004075) DEF8-F	GCGGTGCTCAAGAAGGACTC
(Celaphus_00014788) RCAN3-R	CAGTTCCTCATCTCCCCTCC
(Celaphus_00014788) RCAN3-F	TCTCTCCTGGTCCCAGCTTG
(Celaphus_00016251) TCEA1-R	CAGAATAGCCCTGAAGCAAGAG
(Celaphus_00016251) TCEA1-F	CCCGAGGAAAAGATGAGACG
MERGE.411.1-R	CCACGGCTCAACGACAGATA
MERGE.411.1-F	CAGGACAGGAAGAAGACGAACA
MERGE.629.1-R	TTCTCCCTTACCCCTCACCA
MERGE.629.1-F	AACGATACCAACCAGGACTGC
MERGE.758.2-R	TGAGTAACGCCCACAACCTG
MERGE.758.2-F	TCGGACTGTATTGGAAGCACC
MERGE.8435.1-R	ATGCCTCTTATCACTGCGACTG
MERGE.8435.1-F	TTGACCTTATCCTGGAATGCC
MERGE.16183.1-R	AGCAACAGGGTTACGAAGCC
MERGE.16183.1-F	GCATGATCTCGGGATGGACT
MERGE.19020.1-R	GAGGTGGGGAAGAATAGGGAG
MERGE.19020.1-F	GGGCGCGAGTACAAAGAGA
MERGE.19554.3-R	AAGAATACTGGAGTGGGTTGCC
MERGE.19554.3-F	TCCCTGGTGGTTCAGATGGT
MERGE.21530.1-R	TCCGATCCCGTACCATTAGG
MERGE.21530.1-F	TGGTGGAGAATAAAGAGGCTGA
(Celaphus_00000024) CLDN5-R	GCCTTCCTGGACCACAACA
(Celaphus_00000024) CLDN5-F	CACGGAGTCGTACACCTTGC
(Celaphus_00004417) MED29-R	GTGTATCGGGTCCAGGTTCAG
(Celaphus_00004417) MED29-F	CGCTGCACAGGATCGAAGT
MERGE.10875.2-R	GACGCCGATGGAACTGAGAT
MERGE.10875.2-F	GGTGTAAAGGCACAGCAAAAGA
MERGE.15658.2-R	GTAACATCCTGCGGCTTCCT
MERGE.15658.2-F	ACGAGTGTATTTGGTGGGGG

MERGE.19812.2-R	AGTCAGCGGGCGTTTAGAGT
MERGE.19812.2-F	GCAAGGATGCAAACCAGTCTC
MERGE.4006.4-R	TGAAAGAGGAGTCTGACGCG
MERGE.4006.4-F	TCCTGAGACCATCTTCCCATAG
(Celaphus_00009291) DEPDC7-R	AAGAGTTTGTCAGGCACTTATGG
(Celaphus_00009291) DEPDC7-F	GTGGGTTTTTTGTCTTTTCCG
(Celaphus_00010867) CDC42BPA-R	CTTTGGGGGTCTGTATGTATGAA
(Celaphus_00010867) CDC42BPA-F	GGGGGAAACTGAAACCTCTCT
(Celaphus_00014062) SSPN-R	GGCACCTTCAAACTGTTCCTC
（Celaphus_00014062) SSPN-F	CACATCCTGACACCCACATAAAA
β-actin-R	GTCCGTGACATCAAGGAGAAGC
β-actin-F	AAGGTAGTTTCGTGAATGCCGC
